# Sun Exposure of Body Districts: Development and Validation of an Algorithm to Predict the Erythemal Ultra Violet Dose

**DOI:** 10.3390/ijerph16193632

**Published:** 2019-09-27

**Authors:** Giacomo Salvadori, Davide Lista, Chiara Burattini, Luca Gugliermetti, Francesco Leccese, Fabio Bisegna

**Affiliations:** 1 Department of Energy, Systems, Territory and Constructions Engineering, University of Pisa, 56122 Pisa, Italy; lista.davide@gmail.com (D.L.); f.leccese@ing.unipi.it (F.L.); 2 Department of Astronautical, Electrical and Energy Engineering, Sapienza University, 00184 Rome, Italy; chiara.burattini@uniroma1.it (C.B.); luca.gugliermetti@uniroma1.it (L.G.); fabio.bisegna@uniroma1.it (F.B.)

**Keywords:** sun exposure, solar UV radiation, erythemal UV dose, solar irradiance measurements, human body districts, outdoor workers

## Abstract

Solar Ultra-Violet (UV) radiation has positive and negative effects on human body tissues. Small doses of solar UV radiation are needed by the human skin for the activation of the vitamin D production. Overexposure to solar UV radiation can produce acute and long-term negative effects, such as sunburns and, in the worst cases, cataracts and skin cancers. For this reason, knowing the amount of UV doses received by people is essential to evaluate their risk to UV overexposure and to evaluate the adequate countermeasure to avoid the negative effects. The original contribution of the present study consists in having searched, collected, adapted and processed a series of technical information and analytical relations, developing an algorithm suitable for the calculation of the erythemal UV dose on sloped surfaces exposed to solar radiation, which at the moment is not present in the scientific literature. The results obtained by the algorithm have been compared to the results of a field measurements campaign, carried out in three different Italian sites. Results comparison indicated that measured and calculated values show a sufficient level of agreement, with a mean absolute error equal to 20%.

## 1. Introduction

Solar radiation is a part of the electromagnetic spectrum, mainly emitted in the wavelengths of the infrared (IR), of the visible and of the ultra-violet (UV) [1]. Because of the Earth’s atmosphere, the spectral distribution of the solar radiation is different at the extra-atmospheric level with respect to the sea level. Due to the filtering effect of the stratospheric ozone, the solar radiation at the sea level is mainly distributed in the IR (45%) and in the visible (50%), while the UV radiation covers only the 5% of the Sun’s spectrum [2,3]. Even if the UV radiation represents a minimal percentage of the whole solar radiation reaching the Earth’s surface, it can be considered the main risk factor for human health among the photobiological factors [4,5]. The interaction between UV solar radiation and human tissues may induce several effects, some positive but mostly negative [6]. For example, overexposure to UV radiation may produce acute, chronic and long-term adverse effects on the skin and on the eye, such as sunburns (erythemas), skin ageing, photo-dermatoses and in the most serious cases, skin cancers and cataracts [7,8]. On the other hand, UV radiation has also positive effects for the human body. For example, UV radiation is crucial in vitamin D production and, as consequence, it is important for the prevention of diseases such as osteoporosis and rickets [9]. For UV radiation exposure, the turning point is reaching an exposure level that maintains acceptable production rate of vitamin D, minimizing the adverse risks due to overexposure [10,11].

The effectiveness of UV radiation on human body for the acute effects is accounted by biologic efficiency curves, which represent the spectral weighting functions of the solar radiation on human tissues. The Commission Internationale de l’Eclairage indicated the curve to evaluate the erythemal effects [12]. People’s sensitivity to UV radiation differs on the basis of the personal self-ability defence of the skin. Six skin phototypes, ranging from extremely sensitive (I) to very resistant (VI), were individuated according to the Fitzpatrick scale [13]. For each phototype, the cutaneous response to UV (qualitatively expressed), the cancer risk (expressed on a rising risk scale from +/− to ++++) and the lowest UV dose that causes sunburn (expressed by the Minimal Erythema Dose, MED) are also identified, as summarized in Table 1.

In addition to the MED, which depends on the phototype [1,13], other parameters have been introduced at international level to quantify the risk from exposure to UV radiation for the human skin. The Standard Erythemal Dose (SED) is a parameter introduced in order to define an erythemally-weighted dose equal for all the skin sensitivities [1]. One SED is equivalent to an erythemal radiant exposure of 100 J/m^2^ [1]; it is independent of skin type, and a particular exposure dose in SED may cause erythema in fair skin but none in darker skin. The values of MED can be considered limit values not to be exceeded to ensure the health of workers, while the values of SED are used to assess the actual exposure of workers. For example, a worker with a skin phototype I (extremely sensitive) who is exposed to UV radiation for 1.5 SED reaches his MED, while a worker with a skin phototype VI (very resistant) can be exposed up to 9 SED to reach his relative MED. The UV Index (UVI) is a parameter promoted by the World Health Organization (WHO) to indicate the risk level for skin damage [14]. It is a dimensionless number, ranging from 1 (low) to 11+ (extreme), based on the Erythemal UV irradiance at the ground [15].

People are exposed to UV solar radiation mainly in their own leisure time, but outdoor workers can be exposed to the Sun also during their working activities, and for this reason special attention is posed to this category of people. Some categories of workers (e.g., agricultural and horticultural workers, fishermen, workers in the constructions field, lifeguards, ski resort guides, etc.) can be often overexposed to UV solar radiation, as demonstrated in [16,17]. Given the importance of receiving the right daily dose of UV radiation and in order to reduce the risk of skin damages, it is very important to monitor or to predict the daily UV radiation dose that people receive. For example, if the Erythemal UV dose received by a worker (usually expressed by SED value) is monitored or predicted, it can be compared with the limit values indicated in the literature [18,19], to highlight possible overexposures and take corrective actions in advance.

With reference to the monitoring, personal dosimeters are currently available, they perform direct solar measurements using polysulphone (PS) or polyphenylene oxide (PPO) detectors, which have been calibrated to erythemal exposure [20,21,22,23]. With reference to the analytical prediction, some algorithms for assessing the daily, monthly or yearly erythemal dose in outdoor workers can be found in the literature. Wittlich et al. [24] developed an algorithm for the assessment of the occupational exposure to UV radiation based on time, geographical and personal factors, but with two main limitations: the algorithm provides a retrospective estimation of the solar exposure occurred over a past year, and it requires the use of scientific instruments (such as electronic data loggers) to complete the assessment. The retrospective assessment does not consent to monitor the UV exposure in real time and to prevent overexposure; the use of scientific instruments requires technical knowledge and data post processing. Borra et al. [25] proposed an algorithm for assessing the annual dose of UV solar radiation: it is based on satellite data and personal information acquired by questionnaires; anyway, also in this case, it allows to calculate the dose in the aftermath and not in real time. Park et al. [26] also developed an UV exposure calculation algorithm to support peoples’ daily required UV dose. In this case, the algorithm was used in combination with a UVB LED lighting system and a smartphone’s app, with a very different aim than the overexposure estimation, i.e., to increase the erythemal dose for people who spend too much time indoor environments.

The original contribution of the present study consists in having searched, collected, adapted and processed a series of technical information and analytical relations, developing an algorithm suitable for the calculation of the SED on sloped surfaces (anywhere located on the Earth, anyhow oriented with respect to the south and inclined with respect to the horizontal plane), at the moment not present in the scientific literature. Since the human body districts (i.e., head, torso, arms, legs) can be schematized as a series of prisms, the developed algorithm can be very useful for the assessment of the SED received by the most exposed body surface of a worker engaged in outdoor operations, whatever its posture and orientation are [27]. In this paper, the basic principles used for the development of the algorithm are described together with the calculation method of the Erythemal UV dose on sloped surfaces. Moreover, details are provided for the use of algorithm based on the known input data, and some numerical examples of real cases are shown. Finally, the results of a measurements campaign are shown and discussed in order to provide an experimental validation of the algorithm.

## 2. Algorithm for the Calculation of the Erythemal UV Dose on Sloped Surfaces

The environmental factors are the main elements influencing the exposure to UV radiation of outdoor workers [25,28]. According to the literature [6], the environmental factors that have the most influence on the spectral composition and the amount of UV radiation reaching the Earth’s surface are listed below.

(a) Atmospheric composition: the presence of gaseous or pollutant particles in the Earth’s atmosphere may induce phenomena of absorption, reflection, refraction and/or diffusion of UV radiation. These phenomena influence the amount and the spectral composition of the UV radiation reaching the Earth’s surface. In particular, the stratospheric ozone [29] occurring for the most between 10 and 30 km above the sea level, is able to absorb all the wavelengths lower than 290 nm providing an effective filter function against UV radiation.

(b) Altitude: UV exposure increases of the 8% every kilometre [30,31].

(c) Clouds coverage: UV solar radiation is scattered when passing through the clouds, since they are formed by small water droplets. The effect of clouds is (in general) an attenuation of the UV solar radiation on the surface [32]. Clouds are highly variable in time and space, so there is great difficulty in their specification, and in the quantification of the attenuation effect. This attenuation effect depends on different clouds properties: such as amount, optical thickness, relative position respect to the Sun, their number of layers, etc. Sometimes UV radiation at the ground level may be affected by clouds in such a manner that it may be higher than UV radiation in cloudless conditions. This effect, known as cloud enhancement, is described for example by [33,34].

(d) Reflectance of the ground (or albedo, ρ_UV_): usually in the UV wavelengths, the percentage of radiation reflected by a surface is low (less than the 12%) [35,36], but in some cases, it may be relevant as for example for the fresh snow (ρ_UV_ ≥ 0.80).

**Figure 1:**
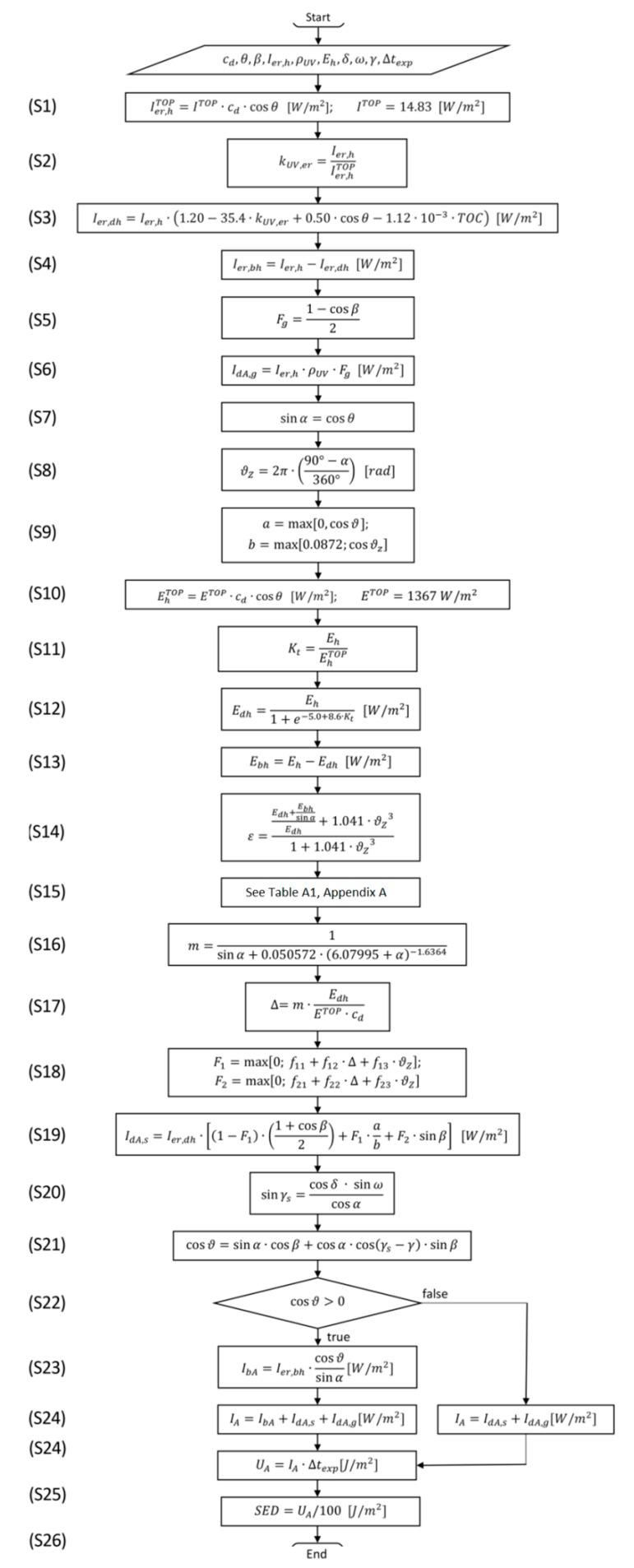
Flow-chart representation of the developed algorithm (the steps are sequentially numbered; for a complete list of used symbols, see Table A2 in Appendix A).

Considered the listed environmental factors, an algorithm for the calculation of the Erythemal UV irradiance on sloped surfaces has been developed (see Figure 1).

The actual UV exposure of a surface is influenced by direct, diffuse and reflected radiation, so it depends on its relative position respect to the Sun, source of the direct component, to the Sky, source of the diffuse component, and to the ground, main source of the reflection. The necessary input data for the developed algorithm are the location of the working site (expressed by latitude and longitude), the inclination angle (β) with respect to the horizontal plane and the azimuth angle (γ) with respect to the south of the considered surface, the Erythemal UV irradiance arriving on a horizontal surface (I_er,h_), the albedo of the ground (ρ_UV_), the time (day and hour) and the duration (Δ_texp_) of the exposure to UV radiation. With the knowledge of the Erythemal UV irradiance on a horizontal surface (I_er,h_), it is possible to calculate its direct and diffuse components according to the method proposed by Reindl et al. [37]. To apply this method to the UV range, the Total Ozone Column (TOC) data (expressed in Dobson Unit, DU) have been considered in the calculations, to take into account its strong impact on the attenuation of the UV radiation [38]. The diffuse (I_er,dh_) and the direct (I_er,bh_) components of I_er,h_ can be evaluated by Equations (1) and (2) respectively [38]:

(1) $$I_{\mathrm{er},\mathrm{dh}}=I_{\mathrm{er},h}\cdot \left(1.20-35.4\cdot k_{\mathrm{UV},\mathrm{er}}+0.50\cdot \cos\theta -1.12\cdot 10^{-3}\cdot \mathrm{TOC}\right)$$

(2) $$I_{\mathrm{er},\mathrm{bh}}=I_{\mathrm{er},h}-I_{\mathrm{er},\mathrm{dh}}$$

where k_UV,er_ is the clearness index for the UV irradiance erythemally weighted, and θ is the solar zenith angle. k_UV,er_ is defined as the ratio between I_er,h_ and the UV irradiance erythemally weighted at the top of the atmosphere, according to Equation (3) [38]:

(3) $$k_{\mathrm{UV},\mathrm{er}}=I_{\mathrm{er},h}/\left(I^{\mathrm{TOP}}\cdot c_{d}\cdot \cos\theta \right)$$

where I^TOP^ is the erythemally weighted UV solar constant equal to 14.83 W/m^2^, and c_d_ is the eccentricity correction factor of the Earth-Sun distance (the mean Earth-Sun distance is 1.495 × 10^11^ m).

The Erythemal UV irradiance arriving on a sloped surface (characterized by an inclination β and an azimuth γ) can be calculated, as the sum of the direct component (I_bA_), the reflected component by the ground (I_dA,g_) and the diffuse component by the sky (I_dA,s_), using Equation (4):

(4) $$I_{A}=I_{\mathrm{bA}}+I_{\mathrm{dA},s}+I_{\mathrm{dA},g}$$

The direct component I_bA_ can be calculated as the product between the direct Erythemal UV irradiance on a horizontal surface and the ratio between the direct irradiance on the sloped surface and the direct irradiance on a horizontal plane, according to Equation (5) [39]:

(5) $$I_{\mathrm{bA}}=I_{\mathrm{er},\mathrm{bh}}\cdot (\cos\vartheta /\sin\alpha )$$

where ϑ is the incidence angle, and α is the height of the Sun on the horizon, for example, calculated according to technical standards on climatic data [40]. The reflected component by the ground I_dA,g_, on the sloped surface, can be expressed by Equation (6) [41]:

(6) $$I_{\mathrm{dA},g}=I_{\mathrm{er},h}\cdot \rho_{\mathrm{UV}}\cdot F_{g}=I_{\mathrm{er},h}\cdot \rho_{\mathrm{UV}}\cdot \left(1-\cos\beta \right)/2$$

where ρ_UV_ is the ground albedo, and F_g_ is the view-factor between the visible portion of the ground and the sloped surface, in the case of a horizontal ground with an unlimited extension and without other reflective surfaces. Typical values of the ground albedo in the UV wavelengths for common materials can be found in the literature [35,36].

The diffuse component by the sky I_dA,s_ can be calculated according to the method proposed by Perez et al. [42,43], considering an anisotropic sky. The Perez-sky model was originally designed to reproduce the diffuse global solar radiation but it has been also applied to reproduce the diffuse Erythemal UV radiation on sloped surface with good results [39,41,44,45]. Once the Erythemal UV irradiance on the sloped surface (I_A_) is known, the radiant exposure (i.e., the energy received by the surface per unit area, U_A_) during the duration of the exposure (Δ_texp_) can be calculated with the Equation (7):

(7) $$U_{A}=I_{A}\cdot \Delta t_{\exp}$$

The SED received by the sloped surface can be calculated with the Equation (8):

(8) $${\mathrm{SED}}_{A}=U_{A}/100$$

The complete algorithm for the calculation of the Erythemal UV dose on sloped surfaces is shown in the form of a flow chart in Figure 1. The algorithm can be applied to the most exposed surface of each body district, considering it as a tilted plane with respect to the ground.

If the diffuse and direct components of the Erythemal UV irradiance (I_er,bh_, I_er,dh_) are known, it is possible to calculate the Erythemal UV irradiance on a sloped surface skipping the first four steps of the algorithm (see steps S1–S4 of Figure 1). In this case, the I_er,h_ and the TOC values are not required among the input data.

## 3. Use of the Algorithm: Numerical Examples

With the aim of discussing the use of the algorithm, numerical examples are shown for the calculation of the Erythemal UV dose on vertical surfaces facing the four cardinal points (case A) and on sloped surfaces facing south and north directions (case B). The calculations are performed for the summer solstice (i.e., 21 June). The Erythemal UV doses are determined for the location of Pisa (43.72° N, 10.39° E, 4.0 m a.s.l.), considering an exposure of one hour, from a half hour before to a half hour after the solar noontime, in the day of the summer solstice and in clear sky conditions. The necessary input data for the calculation of the Erythemal UV dose on the different surfaces are summarized in Table 2. The calculation examples given in the following subsections have the purpose to show the use of the algorithm on surfaces that can be considered as reference. However, the algorithm allows the calculation for surfaces with any orientation (with respect to the south) and inclination (with respect to the horizontal). Since some important body districts can be represented (as a first approximation) as flat surfaces oriented and inclined according to the posture of the worker (e.g., of the front and back of the human torso), important information about the hourly or daily dose received by the worker can be obtained by the use of the algorithm.

### 3.1. Case A: Assessment of Erythemal UV Dose on Different Vertical Surfaces Facing the Four Cardinal Points

Considering the input data indicated in Table 2, the calculation procedure for the Case A starts with the evaluation of the clearness index k_UV,er_ (according to the steps S1 and S2 of Figure 1), and it ends with the calculation of the SED value received by a vertical surface facing one of the four cardinal points (according to the step S26 of Figure 1). The procedure has been applied four times to obtain the SED values for vertical surfaces facing the four different cardinal points. The calculation results obtained with the use of the algorithm are summarized in Table 3. As it can be noticed from Table 3, the Erythemal UV dose values (U_A_) vary from a minimum of 576 J/m^2^ to a maximum of 652 J/m^2^, this latter obtained for the vertical surface facing south. The U_A_ values calculated for the vertical surfaces facing west, east and north are the same; this is in line with the hypotheses of having considered a rather narrow interval of time cantered on the solar noontime, which involves negligible values of direct component of Erythemal UV solar radiation for the vertical surfaces with different exposures from the south and having neglected the presence of reflecting surfaces apart from the horizontal surface of the ground. The SED values obtained from the calculations are obviously proportional to the respective U_A_ values. In particular, if the U_A_ and SED values calculated for the vertical surface facing south are considered, it can be noticed how they are higher than the MED values for phototypes from I to V (see Table 1).

**Figure 2:**
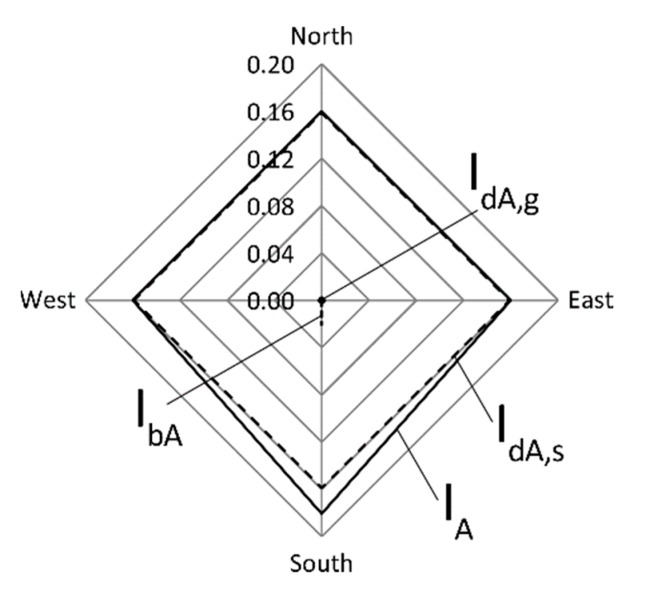
Erythemal UV irradiances received, in the analysed conditions, by vertical surfaces facing the four cardinal points.

With the aim of highlighting the relative weights of the various components that contribute to global Erythemal UV radiation I_A_, according to step S24 of Figure 1, a radar chart is shown in Figure 2.

In the radar chart, the contributions I_bA_, I_dA,s_, I_dA,g_ are indicated for the four analysed vertical surfaces (each of which facing one of the cardinal points). Comparing the values of Table 3 and the chart of Figure 2, it can be observed that the I_dA,g_ values are negligible for all the four vertical surfaces considered, accounting for less than 1% to the respective I_A_ values. The I_dA,s_ values are clearly predominant for all the considered surfaces. For the analysed conditions, the I_bA_ values represent a significant contribution to I_A_ exclusively for the surface facing south, accounting for the 11% to the respective I_A_ value.

### 3.2. Case B: Assessment of Erythemal UV Dose on Different Sloped Surfaces Facing South and North

For the Case B also, considering the input data indicated in Table 2, the calculation procedure follows the steps from S1 to S26 shown in Figure 1. In this case, the Erythemal UV doses are assessed for seven surfaces with different inclination β (with respect to the horizontal plane) from β = 0° to β = 90° with increments of 15° (0°, 15°, 30°, 90°). The procedure has been repeated considering the surfaces facing the south and the north directions. An additional surface with β = 23.4° has been considered for the south exposure because being orthogonal to the Sun direction, it is the one characterized by the maximum values of the direct component of the Erythemal UV irradiance. The calculation results, obtained with the use of the algorithm, are summarized in Table 4 and Table 5 for the surfaces facing south and north directions, respectively. Considering the horizontal surface (β = 0°), the calculation results are indicated only in Table 4, since they do not change with the direction.

Table 4 and Table 5 provide a direct comparison of the U_A_ values obtained for sloped surfaces facing south and north respectively; as it can be noticed, the U_A_ values for the surfaces facing south are on average 1.5 times higher than the respective values for the surfaces facing north with the same β. In fact, in the simulated time conditions, the sloped surfaces facing north are exposed only to reflected and diffuse solar radiation, and the algorithm correctly considers equal to 0 W/m^2^ the direct component (I_bA_). The maximum difference between south and north exposure, in the analysed conditions, is obtained for the sloped surface with β = 15° for which the U_A_ value facing south is 1.9 times higher than the respective value facing north; for this angle of inclination, the reflected component of the ground is minimal. This difference decreases with the increase of β; in fact, for the vertical surface (β = 90°), the U_A_ value facing south is about 1.1 times higher than the respective value facing north. With reference to the U_A_ values for the surfaces facing south, potentially more dangerous for the exposure risks, in the analysed conditions they vary from a minimum of 652 J/m^2^ (obtained for β = 90°) to a maximum of 814 J/m^2^ (obtained for β = 30°). It is interesting to observe that the sloped surfaces facing south, with β till to 60°, have U_A_ values higher than the horizontal surface (β = 0°); for 15° < β < 30° the algorithm considers a higher direct component than for β = 0°, since their inclination is more orthogonal to the Sun position, while for 45° < β < 60° the algorithm results are correctly affected by the reflected component. It can be also observed that the sloped surface with β = 23.4° facing south, despite having the higher value of I_bA_ (0.060 W/m^2^), is characterized by an U_A_ value similar to the one of the surface with β = 30°. However, the U_A_ values for sloped surfaces facing south with β in the range 15°–45° are, in the analysed conditions, very similar (percentage difference lower than 1.4). The SED values obtained from the calculations are, as for the previously discussed Case A, proportional to the respective U_A_ values. In particular, if the U_A_ and SED values calculated for the sloped surfaces facing south are considered, it can be noticed how they are higher than the MED values for phototypes from I to V (see Table 1).

**Figure 3:**
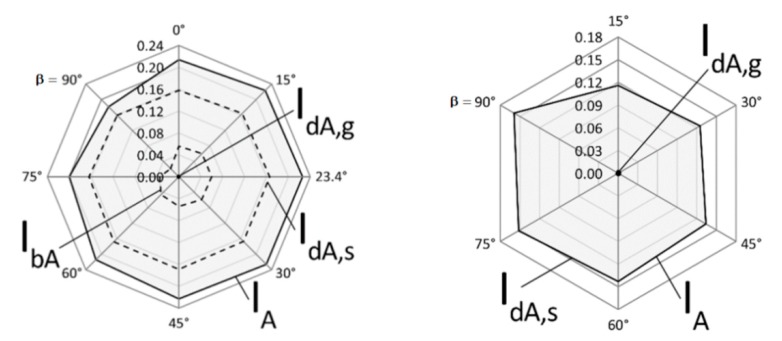
Comparison of the Erythemal UV irradiances received by sloped surfaces facing south (on the **left**) and north (on the **right**).

With the aim of highlighting the relative weights of the various components that contribute to global Erythemal UV radiation I_A_, according to step S24 of Figure 1, radar charts are shown in Figure 3. In the radar charts, the contributions I_bA_, I_dA,s_, I_dA,g_ are indicated for the analysed sloped surfaces facing south (left side chart) and north (right side chart). Comparing the values of Table 4 and Table 5 and the charts of Figure 3, it can be observed that the I_dA,g_ values are negligible for all the four vertical surfaces considered, accounting for less than 1% to the respective I_A_ values. The I_dA,s_ values are clearly predominant for all the considered surfaces. For the analysed conditions, the I_bA_ values represent a significant contribution to I_A_ exclusively for the surface facing south, accounting from the 12% (obtained for β = 90°) till to the 27% (obtained for β = 23.4°) of the I_A_ respective values.

## 4. Validation of the Algorithm

### 4.1. Field Measurements of Erythemal UV Dose

A campaign of field measurements has been carried out for recording the Erythemal UV doses on several sloped surfaces; the results have been used to validate the algorithm. In order to test different conditions, also considering some typical locations where outdoor workers act, the measurements have been performed in three sites having different features and location: the upper terrace of the Faculty of Engineering of Sapienza University in Rome (41.89° N, 12.49° E, 21 m a.s.l.), a parking area in the little town of Guadagnolo located in the centre of Italy (41.91° N, 12.93° E, 1218 m a.s.l.) and a parking area near the School of Engineering in Pisa (43.72° N, 10.39° E, 4 m a.s.l.). The measurements have been carried out in the time band from 11:30 a.m. to 1:30 p.m., which represents the most critical period for the solar radiation exposure.

In Rome, the measurements have been carried out on 21 February, on the upper terrace (floor at 10 m above the ground level) inside the cloister of the ancient building of the Sapienza University. A GigaHertz Radiometer X1-1, placed at a height of 150 cm from the floor, has been used for recording the Erythemal UV irradiance on a horizontal surface (I_er,h_, W/m^2^). The radiometer is equipped with a XD-9506 probe, composed of two different sensors (operating range of wavelengths of 250–325 nm and 325–400 nm respectively), that is able to measure I_er,h_ according to the UV action spectrum [12].

In Guadagnolo, the measurements have been carried out on 25 February, in a parking area (ground level) away from buildings and other reflective surfaces (except the ground). The measurement equipment has been the same used for the measurements made in Rome. In this case (in addition to I_er,h_), values of U_A_ on a horizontal surface, vertical surfaces facing the four cardinal points, sloped (β = 30° and β = 60°) surfaces facing north and south, have been recorded always at a height h = 1.5 m from the ground.

In Pisa, the measurements have been carried out the 16 May 2019, in a park area away from buildings and other reflective surfaces (except the ground). Measurements have been performed with the photo radiometer Delta Ohm HD2102, equipped with a LP471A-UVeff probe. The probe can measure (resolution of 0.001 W/m^2^) the erythemal irradiance (I_er_, W/m^2^) weighted according to the UV action spectrum [12]. The probe is equipped with a diffuser for the correct measure according to the cosine law. The measurement chain (photo radiometer and probe) has been subjected to regular annual calibration (calibration uncertainty lower than 5%) at the manufacturer’s laboratories. The photo radiometer has been used to measure erythemal irradiances on a horizontal surface, a vertical surface facing the four cardinal points, sloped (β = 45°) surfaces facing north and south, always at a height of h = 1.5 m from the ground. By integrating the irradiance values over time, it has been possible to obtain the dose values for all the surfaces.

The recordings have been made with a sample rate of 60 s, evaluating the Erythemal UV dose received by the surfaces every 600 s; these values have been unchanged for the measurements taken in all the sites. The exposure time Δ_texp_ 600 s has been considered adequate for the validation of the algorithm as the variations of the UV irradiance, due to the Sun positions, in this time interval can be considered negligible. The main characteristics of the measurement activity are summarized in Table 6 for the three considered sites. The results of the irradiance values measured in the three sites are shown in Table 7. The Erythemal UV exposure doses have been calculated from the measured irradiance values considering an exposure time Δ_texp_ = 600 s with the relations of steps 25 and 26 of Figure 1.

### 4.2. Application of the Algorithm to the Measured Conditions

By using the developed algorithm and the TOC, E_h_, ρ_UV_ values acquired from the cited references (see Table 2), the U_A_ values have been analytically calculated for all the conditions (site, day, hour, β, γ and Δt_exp_) previously considered for the measurement. The E_h_ values, used as input data for the algorithm, are shown in Table 8. 

For the site of Rome, the U_A_ values have been calculated considering the following input data: n = 52; φ = 41.54° N; ψ = 12.28° E; TOC = 345 DU; ρ_UV_ = 0.057. For the site of Guadagnolo, the U_A_ values have been calculated considering the following input data: n = 56; φ = 41.91° N; ψ = 12.92° E; TOC = 320 DU; ρ_UV_ = 0.018. For the site of Pisa, the U_A_ values have been calculated considering the following input data: n = 136; φ = 43.72° N; ψ = 10.39° E; TOC = 363 DU; ρ_UV_ = 0.055. For the three sites, the values adopted as input data of β and γ were the same of the measurement surfaces (see Table 6 and Table 7). The complete results obtained from the application of the algorithm are shown in Table 9.

### 4.3. Comparison between Measured and Calculated Values of the Erythemal UV Dose

**Figure 4:**
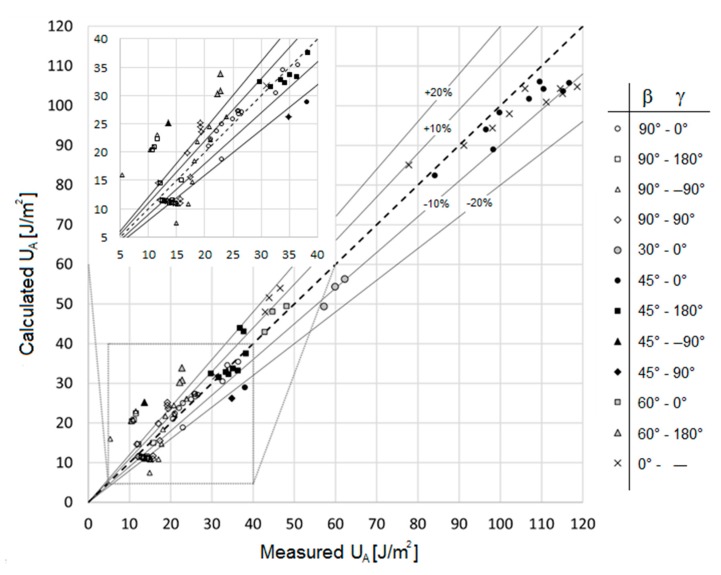
Comparison between measured and calculated Erythemal UV irradiance for the three sites.

**Figure 5:**
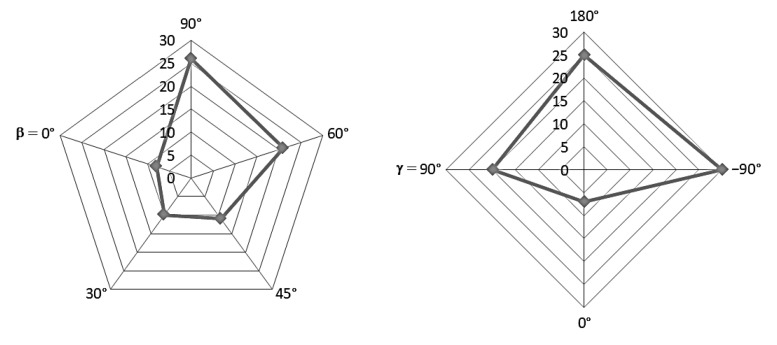
Average values of the percentage deviations, derived by grouping the data (measured and calculated) obtained for the same β values (**left side chart**) and for the same γ values (**right side chart**).

In order to evaluate the accuracy of the algorithm in calculating the Erythemal UV dose for surfaces with different inclinations and directions, the results obtained applying the algorithm have been compared with the measurement results. The comparison, carried out on the basis of the parameter U_A_, is graphically shown in Figure 4; as it can be observed, the measured and calculated data show a sufficient level of agreement, also in light of the complexity of the problem. The value of the mean absolute error, between calculated and measured data, is 20%. The 50% of the measured values are predicted by the algorithm with a deviation lower than 10%, and the 75% of the measured values are predicted by the algorithm with a deviation lower than 18%. It is useful to note that the higher values of the deviations are observed for low values of U_A_ (see Figure 4), for which also the exposure risks are less significant. If only U_A_ values higher than 40 J/m^2^ are considered, the value of the mean absolute error, between calculated and measured data, is 7%. The highest deviations were observed for the measurements taken in the site of Guadagnolo, in particular for the vertical surfaces (β = 90°) facing north and east (γ = 180° and γ = −90° respectively), for which the calculated values are higher than the measured ones. This is probably due to the fact that the forecasting algorithm cautiously does not consider the reductions in the contribution of solar radiation produced by the presence of mountains and hills in the horizon height, more significant in this site with respect to the other analysed sites, which diminished the visible surface of the sky. In Figure 5, average values of the percentage deviations are shown; they were derived by grouping the data (measured and calculated) obtained for the same β values (left side chart) and for the same γ values (right side chart). From the charts of Figure 5, it is possible to observe that the deviations are very low for surfaces with low inclination with respect to the horizontal plane (β ≤ 45°) and for surfaces facing south (γ = 0°), with values of about 10% and 7%, respectively. This is probably due to the fact that in these surfaces the direct component, which is easier to compute respect to the diffuse one, is predominant.

## 5. Conclusions

In this paper, an original algorithm developed for the calculation of the Erythemal UV dose on sloped surfaces (anywhere located on the Earth’s surface, anyhow oriented with respect to the south and inclined with respect to the horizontal plane) has been described, and then it has been applied to some vertical and sloped surfaces facing the different cardinal points. Finally, the algorithm has been validated by comparing the calculated values of Erythemal UV dose (using the algorithm) with those obtained from a measurements campaign in three different sites, located in central Italy.

The numerical examples of the use of the algorithm have been proposed in the paper in order to show the potential of the algorithm. With the algorithm, the values of the Erythemal UV dose and the Standard Erythemal Dose can be estimated in addition to the relative weights of the various components (beam, diffused, reflected) that contribute to global Erythemal UV radiation received by surfaces. The previous values can be calculated for surfaces with any location, orientation and inclination, which can represent different body districts of people. The algorithm provides information of fundamental importance for the estimation of the exposure conditions to solar radiation of outdoor workers. It allows the determination of the worker’s personal exposures, highlighting for example the exceeding of the MED values fixed for a specific phototype, foreseeing any risks of overexposure and allowing to intervene with preventive and protective actions able to reduce the risks of tissue damage.

From the activity conduced for the validation of the algorithm, it has been possible to observe that the measured and the calculated data show a sufficient level of agreement. Considering the differences in the environments of measure, the results show that the algorithm estimates the Erythemal UV dose in good accordance with the field measurements. Assuming the exposure duration of 600 s, as discussed in the paper, the mean absolute error between calculated and measured data is 20%. If only the U_A_ values higher than 40 J/m^2^ are considered, the mean absolute error decreases to 7%; in fact, the higher values of the deviations are observed for low values of U_A_, for which also the exposure risks are less significant. The accuracy of the algorithm could be increased taking into account the reductions in the contribution of solar radiation produced by the presence, in the horizon height, of mountains and hills.

## Figures and Tables

**Table 1:** Skin phototypes and related cutaneous responses to UV, cancer risks and MEDs.

Skin Phototype	Cutaneous Response to UV	Cancer Risk	MED (J/m^2^)
I—Extremely sensitive	Always burns	++++	150
II—Very sensitive	Burns easily	+++/++++	250
III—Sensitive	Burns moderately	+++	300
IV—Mildly sensitive	Burns minimally	++	400
V—Resistant skin	Rarely burns	+	600
VI—Very resistant	Almost never burns	+/−	900

**Note.** Minimal Erythema Dose (MED, J/m^2^) is defined as the least amount of UV radiation that causes reddening and inflammation on a single individual’s previously unexposed skin; the MED value can vary also within the same phototype, on a precautionary basis the lower value for each phototype is given in the Table.

**Table 2:** Required input data for the numerical examples.

TOC = 329 DU	Total Ozone Column (Value from: https://ozoneaq.gsfc.nasa.gov)
β = 90°	inclination angle of the vertical surface (case A)
β = *variable*	from 0° to 90° (case B)
I_er,h_ = 0.214 W/m^2^	Erythemal UV irradiance on a horizontal surface, estimated with the TUV software (https://www2.acom.ucar.edu/modeling/tropospheric-ultraviolet-and-visible-tuv-radiation-model)
δ = 23.45°	solar declination angle (value from [40])
ω = 0°	hour angle (value from [40])
θ = 20.27°	solar zenith angle (value from [40])
ρ_UV_ = 0.011	ground albedo in the UV wavelengths (e.g., green grass, value from [35])
E_h_ = 931.6 W/m^2^	solar total irradiance (value from: http://soda-pro.com/web-services)
γ = −90°, 0°, 90°, 180°	azimuth angles of the surfaces facing east, south, west, north directions respectively
Δt_exp_ = 3600 s	time of exposure

**Table 3:** Calculation results for vertical surfaces facing the four cardinal points (21 June, solar noontime).

		South	West	East	North
I_er,dh_	[W/m^2^]	0.158	0.158	0.158	0.158
I_er,bh_	[W/m^2^]	0.056	0.056	0.056	0.056
I_dA,g_	[W/m^2^]	0.001	0.001	0.001	0.001
I_dA,s_	[W/m^2^]	0.159	0.159	0.159	0.159
cosϑ	-	0.346	0	0	−0.346
I_bA_	[W/m^2^]	0.021	0	0	0
I_A_	[W/m^2^]	0.181	0.160	0.160	0.160
U_A_	[J/m^2^]	652	576	576	576
SED	-	6.5	5.8	5.8	5.8

**Table 4:** Calculation results for sloped surfaces facing the south direction (21 June, solar noontime).

β	[°]	0	15	30	45	60	75	90	23.4
I_er,dh_	[W/m^2^]	0.158	0.158	0.158	0.158	0.158	0.158	0.158	0.158
I_er,bh_	[W/m^2^]	0.056	0.056	0.056	0.056	0.056	0.056	0.056	0.056
I_dA,g_	[W/m^2^]	0	4.01 × 10^−5^	1.58 × 10^−4^	3.45 × 10^−4^	5.89 × 10^−4^	8.72 × 10^−4^	1.18 × 10^−3^	9.72 × 10^−5^
I_dA,s_	[W/m^2^]	0.158	0.164	0.167	0.169	0.168	0.164	0.159	0.166
cosϑ	-	0.938	0.996	0.986	0.908	0.769	0.577	0.346	1.000
I_bA_	[W/m^2^]	0.056	0.059	0.059	0.054	0.046	0.035	0.021	0.060
I_A_	[W/m^2^]	0.214	0.223	0.226	0.223	0.214	0.200	0.181	0.226
U_A_	[J/m^2^]	770	803	814	803	770	720	652	814
SED	-	7.7	8.0	8.1	8.0	7.7	7.2	6.5	8.1

**Table 5:** Calculation results for sloped surfaces facing the north direction (21 June, solar noontime).

β	[°]	15	30	45	60	75	90
I_er,dh_	[W/m^2^]	0.158	0.158	0.158	0.158	0.158	0.158
I_er,bh_	[W/m^2^]	0.056	0.056	0.056	0.056	0.056	0.056
I_dA,g_	[W/m^2^]	2.31 × 10^−3^	2.20 × 10^−3^	2.01 × 10^−3^	1.77 × 10^−3^	1.48 × 10^−3^	1.02 × 10^−3^
I_dA,s_	[W/m^2^]	0.116	0.125	0.134	0.143	0.152	0.159
cosϑ	-	−0.996	−0.986	−0.908	−0.769	−0.577	−0.346
I_bA_	[W/m^2^]	0	0	0	0	0	0
I_A_	[W/m^2^]	0.119	0.127	0.136	0.145	0.153	0.160
U_A_	[J/m^2^]	428	457	490	522	551	576
SED	-	4.3	4.6	4.9	5.2	5.5	5.8

**Table 6:** Characteristics of the measurement activity for the three considered sites.

		Rome			Guadagnolo				Pisa		
Day		21 February 2019			25 February 2019				16 May 2019		
Time band (GMT+1)		11:30–13:00			13:00–13:30				11:30–13:30		
Solar Zenith Angle [°]		58.62–52.75			51.26–51.05				32.95–24.83		
Weather		Sunny			Sunny with some clouds				Sunny		
Type of floor		Red brick			Gravel				Asphalt		
Surface of measure	β [°]	90	45	0	90	60	30	0	90	45	0
	γ [°]	90 0 −90	90 0 −90	-	180 90 0 −90	180 0	0	-	180 90 0−90	180 0	-
	h [m]	1.5									

**Table 7:** U_A_ values obtained from the measurement activity in the three considered sites for horizontal and sloped surfaces facing the four cardinal points.

Site (Day)	Hour	β [°]	γ [°]	U_A_ [J/m^2^]	Site (Day)	Hour	β [°]	γ [°]	U_A_ [J/m^2^]
Rome (2019-02-21)	11:30	90	0	12.96	Pisa (2019-05-16)	12:00	90	180	12.90
	11:45		90	17.40				90	12.96
	12:00		−90	15.31				0	22.02
	12:15	0	-	30.93				−90	20.94
	12:30	45	0	38.02			45	180	34.08
	12:45		90	34.82				0	96.54
	13:00		−90	22.61			0	-	98.10
Guadagnolo (2019-02-25)	13:00	90	180	11.59		12:15	90	180	13.38
			90	19.21				90	13.32
			0	36.42				0	22.98
			−90	11.51				−90	18.72
		60	180	22.71			45	180	33.36
			0	48.11				0	99.78
		30	0	62.27			0	-	102.18
		0	-	46.58		12:30	90	180	14.10
	13:15	90	180	11.10				90	14.34
			90	19.21				0	24.96
			0	33.77				−90	18.12
			−90	10.91			45	180	36.30
		60	180	22.75				0	106.98
			0	44.70			0	-	111.12
		30	0	58.90		12:45	90	180	13.68
		0	-	43.88				90	14.40
	13:30	90	180	10.66				0	25.98
			90	19.37				−90	17.76
			0	32.57			45	180	37.74
			−90	10.30				0	110.52
		60	180	22.20			0	-	115.08
			0	42.79		13:00	90	180	14.82
		30	0	57.22				90	15.66
		0	-	42.96				0	26.58
Pisa (2019-05-16)	11:30	90	180	12.18				−90	17.04
			90	11.82			45	180	35.10
			0	20.58				0	116.70
			−90	23.82			0	-	114.54
		45	180	29.76		13:15	90	180	14.22
			0	84.12				90	15.72
		0	-	77.82				0	25.74
	11:45	90	180	12.42				−90	14.88
			90	12.06			45	180	36.78
			0	21.00				0	109.56
			−90	20.76			0	-	118.68
		45	180	31.68		13:30	90	180	15.78
			0	98.28				90	16.98
		0	-	91.14				0	25.92
								−90	15.18
							45	180	38.28
								0	115.26
							0	-	106.02

**Notes.** The γ values are obviously not indicated for horizontal surfaces (β = 0°). The U_A_ values are referred to an exposure time Δt_exp_ = 600 s.

**Table 8:** E_h_ values of the three considered sites used as input data for the algorithm.

Hour	E_h_ [W/m^2^]		
	Rome	Guadagnolo	Pisa
11:30	160.99	-	851.92
11:45	159.60		874.68
12:00	157.04		894.84
12:15	153.27		911.96
12:30	148.57		925.72
12:45	142.96		935.56
13:00	136.48	100.08	940.20
13:15	-	102.12	922.20
13:30		103.77	911.88

**Table 9:** U_A_ values calculated with the algorithm in the three considered sites for horizontal and sloped surfaces facing the four cardinal points.

Site (Day)	Hour	β [°]	γ [°]	UA [J/m^2^]	Site (Day)	Hour	β [°]	γ [°]	UA [J/m^2^]
Rome (2019-02-21)	11:30	90	0	18.85	Pisa (2019-05-16)	12:00	90	180	11.42
	11:45		90	15.57				90	11.42
	12:00		−90	15.99				0	23.79
	12:15	0	-	31.80				−90	22.23
	12:30	45	0	28.96			45	180	32.26
	12:45		90	26.22				0	94.02
	13:00		−90	25.20			0	-	94.34
Guadagnolo (2019-02-25)	13:00	90	180	22.39		12:15	90	180	11.27
			90	25.15				90	11.28
			0	35.43				0	24.98
			−90	22.99				−90	21.74
		60	180	33.89			45	180	32.82
			0	49.48				0	98.24
		30	0	56.28			0	-	97.95
		0	-	54.00		12:30	90	180	11.11
	13:15	90	180	20.86				90	11.15
			90	24.46				0	25.96
			0	34.54				−90	18.39
			−90	14.86			45	180	33.24
		60	180	30.79				0	101.70
			0	48.07			0	-	100.89
		30	0	54.32		12:45	90	180	10.97
		0	-	51.60				90	11.02
	13:30	90	180	20.44				0	26.67
			90	23.61				−90	14.73
			0	30.51			45	180	43.11
			−90	20.44				0	104.23
		60	180	30.22			0	-	103.02
			0	42.94		13:00	90	180	10.92
		30	0	49.34				90	10.98
		0	-	48.00				0	27.11
Pisa (2019-05-16)	11:30	90	180	14.62				−90	10.89
			90	14.62			45	180	33.72
			0	21.04				0	105.76
			−90	26.14			0	-	104.33
		45	180	32.48		13:15	90	180	11.51
			0	82.41				90	11.70
		0	-	85.05				0	27.28
	11:45	90	180	11.52				−90	7.48
			90	11.52			45	180	43.96
			0	22.37				0	106.05
			−90	24.43			0	-	104.76
		45	180	31.50		13:30	90	180	14.99
			0	88.98				90	19.86
		0	-	89.97				0	27.33
								−90	10.83
							45	180	37.52
								0	103.68
							0	-	104.31

Notes. The γ values are obviously not indicated for horizontal surfaces (β = 0°). The UA values are referred to an exposure time Δtexp = 600 s.

## Appendix A

**Table A1:** Table for the selection of the coefficients f in function of the coefficient of transparency of the sky according to the Perez method (see step S15 of Figure 1).

ε	1.000–1.065	1.065–1.230	1.230–1.500	1.500–1.950	1.950–2.800	2.800–4.500	4.500–6.200	>6.200
*f*_11_	−0.0083	0.1299	0.3297	0.5682	0.8730	1.1326	1.0602	0.6777
*f*_12_	0.5877	0.6826	0.4869	0.1875	−0.3920	−1.2367	−1.5999	−0.3273
*f*_13_	−0.0621	−0.1514	−0.2211	−0.2951	−0.3616	−0.4118	−0.3589	−0.2504
*f*_21_	−0.0596	−0.0189	0.0554	0.1089	0.2256	0.2878	0.2642	0.1561
*f*_22_	0.0721	0.0660	−0.0640	−0.1519	−0.4620	−0.8230	−1.1272	−1.3765
*f*_23_	−0.0220	−0.0289	−0.0261	−0.0140	0.0012	0.0559	0.1311	0.2506

**Table A2:** List of used symbols.

Symbol	Unit	Description
a, b	-	coefficients considering the angle of incidence of Sun rays on the investigated surface
c_d_	-	eccentricity correction factor of Earth’s orbit
E_bh_	W/m^2^	direct component of the solar total irradiance on a horizontal surface
E_dh_	W/m^2^	diffuse component of the solar total irradiance on a horizontal surface
E_h_	W/m^2^	solar total irradiance on a horizontal surface
E^TOP^	W/m^2^	solar total irradiance at the top of the atmosphere, equal to 1367 W/m^2^
F_g_	-	view-factor between the visible portion of the ground and the considered surface
F_1_	-	coefficient for the circumsolar irradiance
F_2_	-	coefficient for the horizon irradiance
I_A_	W/m^2^	Erythemal UV irradiance arriving on the investigated surface
I_bA_	W/m^2^	direct component of Erythemal UV irradiance
I_er,h_	W/m^2^	Erythemal UV irradiance on a horizontal surface
I_dA,g_	W/m^2^	Erythemal UV irradiance reflected from the ground
I_dA,s_	W/m^2^	Erythemal UV irradiance diffused from the sky
I_er,bh_	W/m^2^	direct component of the Erythemal UV irradiance on a horizontal surface
I_er,dh_	W/m^2^	diffuse component of the Erythemal UV irradiance on a horizontal surface
I_er,h_^TOP^	W/m^2^	Erythemal UV irradiance at the top of the atmosphere
I^TOP^	W/m^2^	UV solar constant erythemally weighted, equal to 14.83 W/m^2^
K_t_	-	Clearness index
k_UV,er_	-	UV clearness index
m	-	air optic mass
SED	J/m^2^	Standard Erythemal Dose received by a plane during the time of exposure Δt_exp_
U_A_	J/m^2^	Erythemal UV dose arriving on the investigated surface during an exposure time of Δt_exp_
α	°	angle of elevation of the Sun
β	°	inclination angle (angle between the horizontal and the investigated surface)
γ	°	azimuth angle (angle between the perpendicular projection on a horizontal plane of the investigated surface and the south direction, positive in the direction from south to west)
γ_s_	°	solar azimuth angle
δ	°	solar declination angle
Δ	-	coefficient of brightness
Δt_exp_	s	time of exposure of the investigated surface between the instant t_i_ and the instant t_i+1_
ε	-	coefficient of transparency of the sky
θ	°	solar zenith angle
ϑ_Z_	rad	solar zenith angle
ϑ	°	angle of incidence of the Sun rays on the investigated surface
ρ_UV_	-	ground albedo in the UV wavelengths
ω	°	hour angle
